# Changes in the levels of cytokines, chemokines and malaria-specific antibodies in response to *Plasmodium falciparum *infection in children living in sympatry in Mali

**DOI:** 10.1186/1475-2875-11-109

**Published:** 2012-04-05

**Authors:** Stéphanie Boström, Pablo Giusti, Charles Arama, Jan-Olov Persson, Victor Dara, Boubacar Traore, Amagana Dolo, Ogobara Doumbo, Marita Troye-Blomberg

**Affiliations:** 1 Department of Immunology, Wenner-Gren Institute, Stockholm University, Svante Arrheniusväg 20C, 10691 Stockholm, Sweden; 2 Malaria Research & Training Centre, Faculty of Medicine, Pharmacology & Dentistry, University of Bamako, Bamako, Mali; 3 Division of Mathematical Statistics, Department of Mathematics, Stockholm University, Stockholm, Sweden

**Keywords:** cytokines, chemokines, antibodies, Plasmodium falciparum, Fulani, Dogon

## Abstract

**Background:**

The Fulani are known to be less susceptible to *Plasmodium falciparum *malaria as reflected by lower parasitaemia and fewer clinical symptoms than other sympatric ethnic groups. So far most studies in these groups have been performed on adults, which is why little is known about these responses in children. This study was designed to provide more information on this gap.

**Methods:**

Circulating inflammatory factors and antibody levels in children from the Fulani and Dogon ethnic groups were measured. The inflammatory cytokines; interleukin (IL)-1beta, IL-6, IL-8, IL-10, IL-12p70, tumor necrosis factor (TNF) and the chemokines; regulated on activation normal T cell expressed and secreted (RANTES), monokine-induced by IFN-gamma (MIG), monocyte chemotactic protein (MCP)-1 and IFN-gamma-inducible protein (IP)-10 were measured by cytometric bead arrays. The levels of interferon (IFN)-alpha, IFN-gamma and malaria-specific antibodies; immunoglobulin (Ig) G, IgM and IgG subclasses (IgG1-IgG4) were measured by ELISA.

**Results:**

The results revealed that the Fulani children had higher levels of all tested cytokines compared to the Dogon, in particular IFN-gamma, a cytokine known to be involved in parasite clearance. Out of all the tested chemokines, only MCP-1 was increased in the Fulani compared to the Dogon. When dividing the children into infected and uninfected individuals, infected Dogon had significantly lower levels of RANTES compared to their uninfected peers, and significantly higher levels of MIG and IP-10 as well as MCP-1, although the latter did not reach statistical significance. In contrast, such patterns were not seen in the infected Fulani children and their chemokine levels remained unchanged upon infection compared to uninfected counterparts. Furthermore, the Fulani also had higher titres of malaria-specific IgG and IgM as well as IgG1-3 subclasses compared to the Dogon.

**Conclusions:**

Taken together, this study demonstrates, in accordance with previous work, that Fulani children mount a stronger inflammatory and antibody response against *P. falciparum *parasites compared to the Dogon and that these differences are evident already at an early age. The inflammatory responses in the Fulani were not influenced by an active infection which could explain why less clinical symptoms are seen in this group.

## Background

Infection with *Plasmodium falciparum *remains one of the most common infectious diseases worldwide and is still a major cause of morbidity and mortality in tropical regions, especially in children below the age of five. Immunity to malaria is dependent on both the innate and the adaptive arms (both cell- and antibody mediated) of the immune system, which are required for adequate protection [1].

Inflammatory cytokines play an important role in human immune responses to infection with *P. falciparum *but also seem to contribute to immunopathology and the severity of the disease if not controlled properly. Available data are consistent with a requirement for an early production of in particular interferon (IFN)-γ to mount resistance against infection [2], and protection from clinical episodes [3]. However, other pro-inflammatory cytokines such as interleukin (IL)-12 and tumour necrosis factor (TNF) have also shown to be essential mediators in this protection [4]. They all appear to be necessary for the inhibition of parasite growth and stimulation of phagocytosis to enhance clearance of parasitized erythrocytes. Plasma levels of TNF and nitric oxide, secreted by activated macrophages and neutrophils, are associated with resolution of fever and parasite clearance [3]. Other cytokines such as IL-1, IL-6, IL-8, IL-10 and IL-12 have been implicated in the pathogenesis of severe malaria cases compared to uncomplicated and matched healthy controls [5,6].

Chemokines are chemotactic cytokines that play important roles in bridging the innate and the adaptive immune system [7]. They orchestrate the migration of leucocytes and other cells by activating corresponding receptors on responsive cells, thereby inducing chemotaxis of immune cells to sites of infection. Studies have shown that chemokine levels in children with acute *P. falciparum *infections vary with disease severity [8] and some, such as IP-10 and RANTES, have been associated with mortality during cerebral malaria [9-11].

Even though the innate immune system is essential during *P. falciparum *infections, the importance of antibody-mediated response against *P. falciparum *parasites was shown 50 years ago in passive transfer studies, in which immunoglobulin (Ig) G antibodies from immune African adults reduced the level of parasites in Gambian children [12]. However, accumulating evidence suggests a role for the cytophilic antibodies IgG1 and IgG3 to be protective against *P. falciparum *infection, whereas the role for IgG2 and IgG4 in terms of protection are less clear and has been suggested to interfere with the protective mechanisms seen by the cytophilic antibodies [13-16]. Thus, protection against malaria depends on early, and intense but carefully balanced pro-inflammatory response to control/reduce the parasite numbers in the human host, which in turn shape the following adaptive immune response, and when such is properly induced it will protect against challenge.

In Mali, previous studies have shown different susceptibility to *P. falciparum *infection between two ethnic groups; the Fulani and the Dogon [17]. Similar findings have been shown in Burkina Faso between Fulani and their sympatric ethnic groups Rimaibé and Mossi [18]. These populations live under similar social, cultural and geographic conditions and are exposed to similar malaria pressure [17,18]. Despite this, the Fulani are more resistant to *P. falciparum *infection than the other ethnic groups as reflected by fewer clinical symptoms of malaria and less parasite clones and numbers found in their blood [17,18]. In addition, the Fulani have also been shown to have higher titres of malaria-specific IgG [15,16,19] and IgM [14] antibodies as well as higher total IgG and IgM levels than other sympatric groups [14]. This higher humoral response in the Fulani has been associated with increased splenomegaly compared to neighbouring ethnic groups [20]. The consistently increased spleen rates found in the Fulani compared to the Dogon (whose spleen rates change upon infection) suggest a more acute response in the spleen to the parasites and clinical disease in the Dogon. Thus, the two groups appear to have different responses when coping with malaria infections. Furthermore, the Fulani has been shown to have higher percentages of IL-4 and IFN-γ producing cells [19] and, recently, lower expression of genes distinctive of Treg activity (foxp3 and CTLA-4) has been observed indicative of a stronger inflammatory response [21]. Established genetic malaria resistance factors such as haemoglobin S and C, alpha thalassemia, G6PD and HLA B have been shown to occur at a lower frequency in the Fulani than in their sympatric neighbours [22], suggesting that various hemoglobinopathies is not the reason for Fulani's lower susceptibility to malaria. The Fulani seem to mount a more potent early IFN-γ response against the parasite [23] compared to other sympatric ethnic groups, which indicates differences in immune regulation between these groups. This is emphasized by the findings that Fulani and Dogon children respond differently to *P. falciparum *infection in terms of antigen-presenting cell (APC) subset activation and toll-like receptor (TLR) stimulations [24]. Nevertheless, the mechanism underlying the lower susceptibility to *P. falciparum *infection in Fulani as compared to other sympatric ethnic groups is not fully understood. Earlier studies have mostly focused on asymptomatic adults, and little is known about this in children. The focus of this study has, therefore, been to investigate inflammatory- and antibody responses in infected and uninfected children from Dogon and Fulani ethnic groups living in Mali, by measuring selected cytokines, chemokines and IgG subclasses in plasma obtained from these children.

## Methods

### Study population

Children between 2 and 10 years of age belonging to either the Fulani or the Dogon ethnic group was included in the study. In total, 40 children from the Dogon and 37 from the Fulani ethnicity were recruited. Healthy children and children diagnosed with asymptomatic malaria were included from both ethnic groups. A thick blood smear was made from each donor and the slides were stained in 3% Giemsa and examined for the presence of *P. falciparum *parasites. Malaria infection was defined as having a positive thick blood smear with or without any malaria symptoms. Axillary temperature was measured in all children and symptomatic malaria was defined as fever ≥ 37.5°C, or history of fever, plus the presence of any density of parasites in the blood. Among the Dogon, 20 children were undergoing a malaria infection and 20 children were healthy, while in the Fulani, 14 children were having a malaria infection and 23 were healthy. Written informed consent was obtained from both the community and from the children's guardians before inclusion in this study. The study was approved by the institutional review boards of the University of Bamako Mali (N°08_64/FMPOS).

### Study area

This study was conducted in a rural area of the Dogon valley located approximately 850 km from the capital of Bamako, in Mali. The malaria transmission in the area is meso-endemic with intense transmission during the rainy season that usually extends from June to October [17]. Samples were collected from October to November 2008, from two rural villages (Manteourou and Binédama) where people from the Dogon and Fulani ethnic groups live together in sympatry. The study area and the study population have been described in detail elsewhere [17].

### Sample collection

Blood samples were collected from uninfected and infected children of both ethnicities. From each donor, 6 ml of venous blood were collected in heparin tubes (BD Vacutainer^® ^Plasma Tube, 143 USP Units of Sodium Heparin freeze dried) and plasmas were obtained by centrifugation and were kept at -80°C and transported to Stockholm University and analysed for cytokine and antibody levels.

### Determination of cytokine levels in plasma samples using ELISA

To quantify levels of IFN-α and IFN-γ in plasma, ELISA kits using pairs of capture and detection antibodies were used according to the manufacturer's recommendation (Mabtech, Stockholm, Sweden). All samples were diluted 1:2 and tested in duplicates. The enzyme-substrate reaction was developed using p-nitrophenyl phosphatase (Sigma, St Louis, MO, USA) and the optical densities were measured at 405 nm in an ELISA plate reader (VmaxTM Kinetic Microplate Reader, Menlo Park, CA, USA). The concentrations were calculated from a standard curve obtained in a sandwich ELISA with eight dilutions of lyophilized native human IFN-α standard (range 3.0-10,000 pg/ml) and recombinant human IFN-γ standard (range 3,000-1.0 pg/ml).

### Determination of cytokines and chemokines in plasma samples using cytometric bead array

Levels of IL-1β, IL-6, IL-8, IL-10, IL-12p70, TNF, regulated on activation normal T cell expressed and secreted (RANTES), monokine-induced by IFN-γ (MIG), monocytes chemotactic protein (MCP)-1 and IFN-gamma-inducible protein (IP)-10 were measured in plasma using cytometric bead array (CBA) (BD Biosciences, San Diego, CA, USA) according to the manufacturer's recommendation. Briefly, 24 μl of bead populations with discrete fluorescent intensities coated with cytokine capture antibodies, were added to 24 μl of plasma sample and the mixtures were incubated for 90 min in room temperature. At the same time, standards for each cytokine/chemokine (range 0-10.000 pg/ml) were also mixed with cytokine specific capture beads. After incubation, the samples and standards were washed to remove unbound material and 24 μl of phycoerythrin (PE)-conjugated anti-human inflammatory cytokine or chemokine antibodies were added and mixtures were incubated again for 90 min followed by washing an acquisition on a fluorescent activated cell sorter (FACS) Calibur (BD Biosciences). Samples and standards were analysed using FCAP Array software v1.0.1 (BD/Softflow, Pécs, Hungary). To determine levels of IL-1β, IL-6, IL-8, IL-10, IL-12p70 and TNF samples were incubated with anti-cytokine capture beads for 90 min, washed and incubated with PE-detection reagent for another 90 min and after final washing acquisition was done on FACS. Calibration was performed on the flow cytometer before acquisition using BD FACSComp™ and BD CaliBRITE™. The lower detection limit for the various cytokines and chemokines was 3.6, 7.2, 2.5, 3.3, 3.7, 1.9, 1.0, 2.5, 2.7 and 2.8 pg/ml for IL-8, IL-1β, IL-6, IL-10, TNF, IL-12p70, RANTES, MIG, MCP-1 and IP-10 respectively.

### Preparation of crude malaria antigen

*Plasmodium falciparum *parasites of the strain F32 were held in continuous cultures as described elsewhere [25] and kept synchronized by treating the parasites repeatedly with sorbitol. Crude malaria antigen was prepared as previously described by Troye-Blomberg and colleagues [26]. A control antigen was prepared the same way as the crude malaria antigen by sonication of erythrocytes that had been held in cultures and had been treated the same way as the parasite cultures.

### Determination of circulating levels of malaria-specific antibodies

Levels of malaria-specific antibodies were quantified using sandwich ELISAs as described by Bolad and colleagues [14] but with some modifications. Briefly, 96-well ELISA plates (Costar, Corning, NY) were coated with 50 μl crude malaria antigen (10 μg/ml) in coating buffer (pH 9.6) over night at 4°C. After blocking for 2 hr with 100 μl of coating buffer containing 0.5% bovine serum albumin, the plates were washed with phosphate buffered saline containing 0.05% Tween 20. Plasma dilutions for the determination of the IgG subclasses (50 μl, 1:20 for IgG2 and IgG4 and 1:400 for IgG1 and IgG3) were added to the plates and incubated for 1 hr at 37°C. For determination of total malaria-specific IgG and IgM antibodies, plasma samples were diluted 1:1000 and 1:500, respectively. To detect IgG2, IgG3 and IgG4, 50 μl biotin-conjugated mouse anti-human monoclonal antibodies (IgG2 diluted 1:3000 (PharMingen, San Diego, CA, USA), IgG3 diluted 1:1000 (Caltag laboratories, Burlingame, CA, USA) and IgG4 diluted 1:2000 (PharMingen), and strep-ALP (Mabtech, Stockholm, Sweden) were used for each subclass. Detection of IgG1 was done using a mouse anti-human monoclonal IgG1 antibody (SkyBio, Bedfordshire, England) and alkaline phosphatase conjugated goat anti-mouse Ig (Dako Denmark A/S, Glostrup, Denmark). To detect malaria-specific IgG and IgM, 50 μl goat-anti-human IgG-ALP (Mabtech, räcker med stad och land första gången företaget nämns sedan bara namnet på företaget) and 50 μl goat-anti-human IgM-ALP (Jackson ImmunoResearch Laboratories, PA, USA) were added to the wells. The assays were developed using p-nitrophenyl phosphatase as a substrate (Sigma, St Louis, MO, USA) and the optical densities were measured at 405 nm in an ELISA plate reader (VmaxTM Kinetic Microplate Reader, Menlo Park, CA, USA). The concentrations of malaria-specific antibodies were calculated from standard curves obtained from sandwich ELISA with six dilutions of myeloma proteins of the IgG1-4 isotypes (Biogenesis, Poole, England) or for total malaria-specific IgG and IgM antibodies, using highly purified IgG or IgM antibodies, respectively (Jackson ImmunoResearch Laboratories, PA, USA).

### Statistical analysis

Differences between the Dogon and Fulani were analysed for statistical significance using Kruskal-Wallis non-parametric test. If differences were seen, a Mann-Whitney U non-parametric test was performed, to distinguish intra- and inter ethnic differences between infected and non-infected Fulani and Dogon. Spearman rank correlation was employed when analyzing possible correlations. Multiple regression was employed to correct for age on antibody titres. Statistical significance was assumed when p < 0.05. The data were analysed using StatView software 5.0.1 and Stata 12.

## Results

### Study subject characteristics

The results from study subject characteristics have been described elsewhere [24]. There were no differences in age, haemoglobin levels and axillary temperature between the Dogon and Fulani children groups, irrespective of infectious status (Table 1). Likewise, no differences were seen in haemoglobin and axillary temperature between uninfected individuals from both ethnic groups. However, when children were divided into infected and uninfected individuals, infected Dogon children had significantly higher axillary temperature compared to uninfected Dogon children (37.74°C versus 36.43°C, p < 0.0001) and to infected Fulani children (37.74°C versus 36.81°C, p = 0.002). There was no difference between infected and uninfected Fulani children (36.81°C versus 36.71°C, p = 0.97). The infected Fulani children were found to be younger than both the infected Dogon (4.50 versus 6.70, p = 0.01) and the uninfected Fulani children (4.50 versus 6.44, p = 0.007). No difference in age was seen between infected and uninfected Dogon children (6.70 versus 6.35, p = 0.47). The mean parasite density was 24,151 (100-122,000) asexual parasites/μl for the Fulani and 13,692 (575-48,625) asexual parasites/μl for the Dogon (p = 0.17). There were two outliers among the infected Fulani children with very high parasitaemia. When these were excluded from the analysis the mean parasite density in Fulani was 1,618 (100-7,875) asexual parasites/μl and hence being less parasitized compared to Dogon (p = 0.004).

**Table 1:** Description of the Fulani and the Dogon children in the present study

n = 77	Mean Age (Year)	Mean Hb (g/dL)	Mean axillary temperature (°C)	Mean Parasitaemia (min-max) (parasites/μl)	No of individuals
**Uninfected Dogon**	6.35	10.36	36.43	-	20

**Infected Dogon**	6.70	9.80	37.74	13692 (575-48625)	20

**Uninfected Fulani**	6.44	9.78	36.71	-	23

**Infected Fulani**	4.50	9.36	36.81	27537 (100-122000)	14

***P *value**	*DI-FI, *F-FI	NS	*D-DI, *DI-FI	NS	-

**All Dogon**	6.53	10.09	37.08	-	40

**All Fulani**	5.70	9.62	36.75	-	37

***P *value**	NS	NS	NS	-	-

D: uninfected Dogon, DI: infected Dogon, F: uninfected Fulani, FI: infected Fulani. *P value ≤ 0.01. Mann-Whitney *U *test. NS: not significant.

### Increased plasma cytokine levels in Fulani children as compared to Dogon

**Figure 1:**
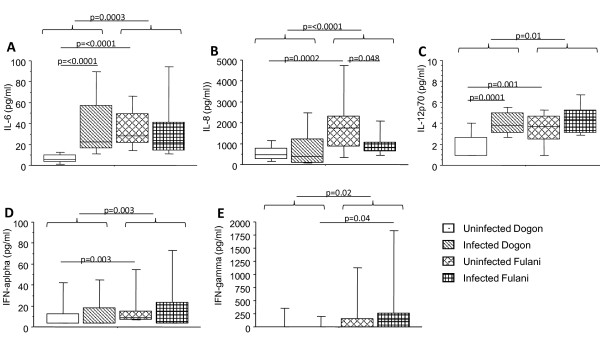
**Levels of inflammatory cytokines in plasma from Dogon and Fulani children**. Blood plasma samples from 77 children were analysed for circulating cytokines. Levels of IL-6 (A), IL-8 (B) and IL-12p70 (C) were measured with cytometric bead array. Levels of IFN-α (D) and IFN-γ (E) were measured with ELISA kits. The children were subdivided in terms of ethnicity and with regard to being infected or not according to slide positivity to malaria; i.e. uninfected Dogon (n = 20), infected Dogon (n = 20), uninfected Fulani (n = 23) and infected Fulani (n = 14). The boxes represent the values between 25% and 75% quartile and the line indicates the median. The whiskers indicate the 10% and 90% percentiles. Statistical analysis was done by Mann-Whitney *U *test.

Blood plasma samples from the Dogon and Fulani children were analysed for levels of IL-1β, IL-6, IL-8, IL-10, IL-12p70, TNF, IFN-α and IFN-γ (Figure 1). Detectable levels of each cytokine were divided according to infectious status and/or ethnicity. When comparing the Fulani to the Dogon children regardless of infectious status, higher levels of IL-6 (p = 0.0003; Figure 1A), IL-8 (p = < 0.0001; Figure 1B), IL-12p70 (p = 0.01; Figure 1C), IFN-α (p = 0.003; Figure 1D) and IFN-γ (p = 0.017; Figure 1E) were found in the Fulani. When comparing the uninfected children from both groups with each other, higher levels of IL-6 (p = < 0.0001), IL-8 (p = 0.0002), IL-12p70 (p = 0.001) and IFN-α (p = 0.003) were found in Fulani compared to Dogon children.

When comparing the levels in the infected children of the two groups, IFN-γ was the only factor out of all tested that showed a significant difference, where the levels were higher in infected Fulani as compared to infected Dogon (p = 0.04).

When the levels were compared within the same ethnic group in relation to *P. falciparum *infection, it was found that infected Dogon children had significantly higher levels of IL-6 (p = < 0.0001) and IL-12p70 (p = 0.0002) compared to the uninfected Dogon. Likewise, the only factor that showed significant difference between infected and uninfected Fulani was IL-8, which was lower in the infected Fulani (p = 0.048).

In most cases levels of IL-1β and TNF were under the detection limit for the kit and were therefore excluded from analysis.

### Chemokine levels differ in infected Dogon and Fulani children

**Figure 2:**
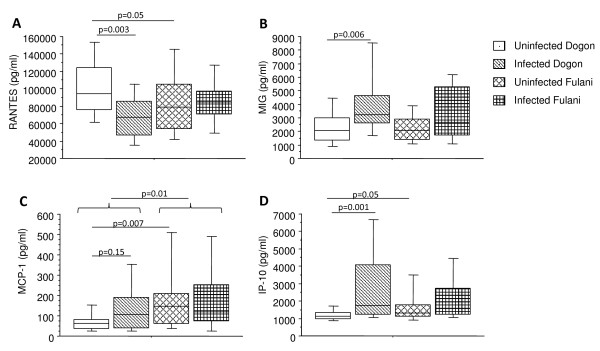
**Levels of chemokines in plasma from Dogon and Fulani children**. Blood plasma samples from 77 children were analysed for circulating chemokines. Levels of RANTES (A), MIG (B), MCP-1 (C) and IP-10 (D) were measured with cytometric bead array. The children were subdivided in terms of ethnicity and with regards to being infected or not according to slide positivity to malaria; i.e. uninfected Dogon (n = 20), infected Dogon (n = 20), uninfected Fulani (n = 23) and infected Fulani (n = 14). The boxes represent the values between 25% and 75% quartile and the line indicates the median. The whiskers indicate the 10% and 90% percentiles. Statistical analysis was done by Mann-Whitney *U *test.

Blood plasma from the Dogon and Fulani children were analysed for levels of RANTES, MIG, MCP-1 and IP-10 (Figure 2). When comparing the Fulani children to the Dogon children, regardless of infectious status, MCP-1 but not RANTES, MIG and IP-10 showed significant difference between the two ethnic groups (p = 0.01; Figure 2C).

When comparing the uninfected children from both groups, the uninfected Dogon children had higher levels of RANTES (p = 0.05) but lower levels of MCP-1 (p = 0.007) and IP-10 (p = 0.05) compared to uninfected Fulani. In contrast, when comparing infected Dogon to infected Fulani children, no such difference was seen for any of the factors tested.

When comparing children from the same ethnic group, there were no differences for any of the factors tested between infected and uninfected Fulani children. In contrast, infected Dogon children had significantly lower plasma levels of RANTES (p = 0.003) and significantly higher plasma levels of both MIG (p = 0.006) and IP-10 (p = 0.001) as compared to uninfected Dogon. The infected Dogon also had increased levels of MCP-1 compared to uninfected Dogon, even though this did not reach statistical significance (p = 0.15).

### Malaria-specific antibody titres are higher in Fulani children as compared to Dogon

**Figure 3:**
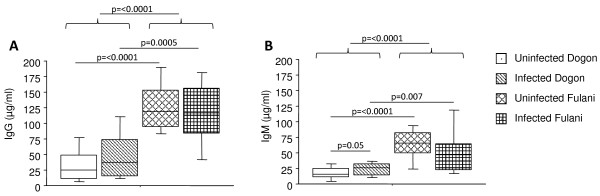
**Distribution of malaria-specific IgG and IgM antibodies in response to *Plasmodium falciparum *infection in Fulani and Dogon children**. Blood plasma levels from 77 children were analysed for malaria-specific IgG (A) and IgM (B) antibodies. The children were subdivided in terms of ethnicity and with regards to being infected or not according to slide positivity to malaria; i.e. uninfected Dogon (n = 20), infected Dogon (n = 20), uninfected Fulani (n = 23) and infected Fulani (n = 14). The boxes represent the values between 25% and 75% quartile and the line indicates the median. The whiskers indicate the 10% and 90% percentiles. Statistical analysis was done by Mann-Whitney *U *test.

**Figure 4:**
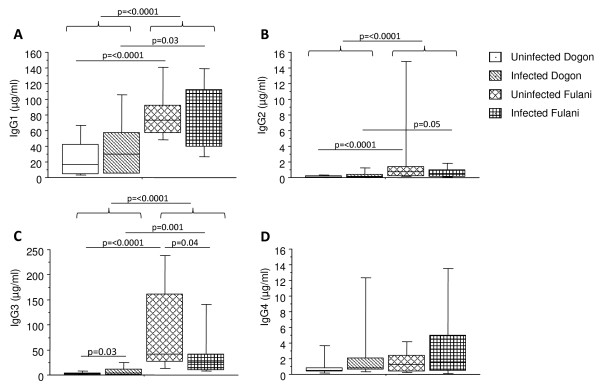
**Distribution of malaria-specific IgG subclasses in response to *Plasmodium falciparum *infection in Fulani and Dogon children**. Blood plasma samples from 77 children were analysed for malaria-specific IgG subclasses IgG1 (A), IgG2 (B), IgG3 (C) and IgG4 (D). The children were subdivided in terms of ethnicity and with regards to being infected or not according to slide positivity to malaria; i.e. uninfected Dogon (n = 20), infected Dogon (n = 20), uninfected Fulani (n = 23) and infected Fulani (n = 14). The boxes represent the values between 25% and 75% quartile and the line indicates the median. The whiskers indicate the 10% and 90% percentiles. Statistical analysis was done by Mann-Whitney *U *test.

Levels of malaria-specific total IgG and IgM as well as the IgG subclasses; IgG1, IgG2, IgG3 and IgG4 antibodies were measured in blood plasma samples from Dogon and Fulani children (Figure 3). When analysing the data regardless of infectious status, the Fulani children exhibit higher titres of both malaria-specific IgG and IgM antibodies as compared to the Dogon population (p ≤ 0.0001; Figure 3A + B). When comparing infected and uninfected children within the same ethnic group, infected Dogon children had higher IgM titres as compared to uninfected Dogon (p = 0.05). In concordance with this, higher titres of malaria-specific IgG subclasses in Fulani population as compared to Dogon for IgG1 (p ≤ 0.0001; Figure 4A), IgG2 (p ≤ 0.0001; Figure 4B) and for IgG3 (p ≤ 0.0001; Figure 4C) but not for IgG4 (p = 0.19; Figure 4D) were found. The same differences were also seen when only comparing the uninfected individuals from the two ethnic groups.

When comparing the infected children of both ethnicities, the infected Fulani children had higher titres of malaria-specific IgG1 (p = 0.03) and IgG3 (p = 0.001) as compared to infected Dogon children (Figure 3A + C).

When comparing infected and uninfected children within the ethnic groups, IgG3 was significantly higher in infected Dogon as compared to uninfected individuals (p = 0.034). The reverse pattern was seen in Fulani, in which uninfected Fulani had higher titres as compared to infected individuals (p = 0.045) (Figure 3C). No antibody reactivity for any of the groups was seen for the control antigen (not listed).

## Discussion

Available studies trying to resolve the reason for the Fulani's relative protection against malaria have so far focused on immunological responses mostly in adults and not so much is known about these responses in children. The previously established relative protection for Fulani seen in adults was in this study shown to be evident during early childhood in this ethnic group. Children from the Fulani ethnic group had higher titres of malaria-specific antibodies and pro-inflammatory cytokine levels as well as unchanged chemokine levels upon an infection, as compared to Dogon children, suggesting that these differences are established already early in life.

Cytokines and chemokines are essential mediators during *P. falciparum *infection and the balance between pro- and anti-inflammatory cytokines may be important for the clinical outcome of malaria [27]. Higher levels of all detectable cytokines were found in the Fulani group when comparing all Fulani children to all Dogon children, irrespective of infectious status. When children were further subdivided and the uninfected children from both ethnic groups were compared, the uninfected Fulani were found to have higher levels of IFN-α, IL-6, IL-8 and IL-12 compared to the uninfected Dogon. This indicates that Fulani children might be more prone to combat *P. falciparum *infections, possibly due to higher baseline levels of these cytokines.

When comparing the infected children from both ethnic groups, IFN-γ was the only factor out of all tested that was significantly increased in infected Fulani compared to infected Dogon. This observation is in line with previous findings by McCall and colleagues who reported that mononuclear cells from Fulani are able to produce a 10-fold stronger IFN-γ response after stimulation with *Plasmodium *parasites compared to cells from Dogon [23]. Available studies in both humans and mice have demonstrated the importance of an early IFN-γ response as a crucial determinant in the outcome of the infection and the well-being of the patient [28,29]. The result suggests that the increased levels of IFN-γ seen in infected Fulani may play a critical role in the difference in susceptibility to malaria in these two ethnic groups.

When comparing the uninfected children from both ethnic groups, the levels of IL-8 were found to be higher in uninfected Fulani compared to uninfected Dogon. However, when comparing differences within the same ethnic group, the levels of IL-8 were significantly lower in infected Fulani compared to their uninfected peers and this difference was not evident between infected and uninfected Dogon. IL-8 is a chemoattractant cytokine known to be involved in recruiting neutrophils to inflammatory sites. Very little information concerning the role for IL-8 in malaria pathogenesis/protection exists. IL-8 has been shown to be elevated in a small group of severe malaria cases in adults [30]. In addition, high expression levels of IL-8 mRNA have also been demonstrated in placental malaria [31]. Thus, the relevance for the lower levels of IL-8 that Fulani children exhibit upon infection is presently unknown and needs to be further investigated.

Further, the only cytokines that showed differences between infected and uninfected Dogon were IL-6 and IL-12p70. The results showed higher levels of IL-6 and IL-12p70 in infected Dogon compared to their uninfected counterparts, and these differences were not seen between infected and uninfected Fulani children. IL-6 is a major mediator of the acute phase response. Increased *P. falciparum*-induced IL-6 production has previously been associated with increased incidence of clinical episodes [32] and others have suggested IL-6 to be a marker for complicated *P. falciparum *malaria [33]. The fact that IL-6 increased upon infection in Dogon but not in Fulani, might imply this cytokine to be important in the pathogenesis of malaria. IL-12p70 has a fundamental role in the induction of Th1-associated immunity. Low levels of IL-12p70 have been reported to be associated with severe malaria disease in children [34,35]. The Fulani children had unchanged levels of IL-12p70 upon infection, while the levels increased in Dogon children. However, there were no correlations between the levels of IL-6 (p = 0.24, Rho = 0.28) and IL-12p70 (p = 0.26, Rho = -0.28) with parasitaemia, which could be due to small sample size in this study population.

The role of chemokines and their distribution in plasma has not been investigated in the Fulani and Dogon ethnic groups. The results from this study show that all chemokines tested were unchanged upon an infection in Fulani children, while this was not the case in Dogon. Infected Dogon children undergoing an infection had lower levels of RANTES compared to uninfected Dogon. Low levels of RANTES have previously been reported to be associated with mortality in children with severe malaria [36], in particular in cerebral malaria [9], and decreased levels have also been reported to be correlated with increased disease severity [8]. One reason for the low levels of RANTES in severe malaria may be due to thrombocytopaenia that is often seen during these conditions, as platelets are a major reservoir of RANTES. The low level of RANTES seen in infected Dogon could thus be a result of destroyed platelets even though this was not tested.

The observed higher levels of MIG, MCP-1 and IP-10 in infected Dogon children compared to uninfected children. This marked up-regulation of chemokines was not evident in the infected Fulani children. Chemokines are involved in attracting different immune cell populations to the site of infections thereby aiding in the inflammatory response. Some of these factors have been implicated in severe malaria disease as well as in inflammatory diseases [37]. High levels of IP-10 have been shown to be associated with increased risk of cerebral malaria associated mortality [10], IP-10 and MIG are required for development of murine cerebral malaria [38] and elevated levels of these factors have also been found in visceral leishmaniasis [39].

Taken together it seems Fulani already have high baseline levels of these chemokines in circulation compared to Dogon since the levels do not change when they become infected, again suggesting that they are more prepared for combating infections. Since chemokines such as MIG, MCP-1 and IP-10 are involved in cell migration, the lower frequencies of these chemokines found in uninfected Dogon might imply that these cells are not able to migrate as well as cells from uninfected Fulani. Interestingly, Arama and colleagues [24] reported that infected Dogon children have higher percentages of certain antigen-presenting cell (APC) subsets in circulation but that they are less activated compared to APC obtained from Fulani children. In contrast, the Fulani children had fewer but more activated APC in the circulation, which suggests that the cells have migrated to the secondary lymphoid organs and are therefore not seen in the circulation.

It has been known since the early 1960s that immunoglobulins (Ig) play an important role in malarial immunity [12]. The result from this study indicates that the Fulani children have a stronger anti-malaria specific antibody response as compared to the Dogon, as revealed by higher titres of malaria-specific IgG and IgM antibodies. This has been reported earlier by others who compared adult Fulani with adults from their neighbouring ethnic groups, living in both Burkina Faso [14] and in Mali [14-16]. Previous studies have shown that antibody titres are correlated with age and parasitaemia [40], therefore the relationship between high parasitaemia and low antibody titres as well as a correlation between age and high antibody titres in these children was investigated. However, such analysis showed that there were no correlations for total IgG in infected Dogon (p = 0.63, Rho = 0.11) as well as for infected Fulani (p = 0.43, Rho = -0.32) between high parasitaemia and antibody titres. In contrast, the correlation between age and antibody titres showed an increase of total IgG antibodies with increasing age in uninfected Dogon (p = 0.005, Rho = 0.64), which was not the case in uninfected Fulani (p = 0.47, Rho = -0.17), suggesting that Fulani children already have high antibody titres irrespective of age in this study population. Similar results were found for IgG1 (p = 0.07, Rho = 0.40) and IgG3 (p = 0.02, Rho = 0.52) between age and antibody titres in the uninfected Dogon, which was not the case for uninfected Fulani (p = 0.57, Rho = -0.14) and (p = 0.23, Rho = 0.25) for IgG1 and IgG3, respectively.

To reassure that the malaria-specific antibodies are specific for the parasite, a control antigen of sonicated erythrocytes were included on all plates. There was no reactivity in any sera tested with sonicated erythrocytes confirming that the antibodies detected against the crude antigen are parasite specific.

Growing evidence supports that not all immunoglobulin subclasses are associated with protection against malaria, but rather it is the cytophilic subclasses; i.e. IgG1 and IgG3, that are important. IgG1 and IgG3 antibodies have shown to be crucial in parasite clearance by binding to the surface of the infected erythrocyte or to mediate opsonization by cooperating with human monocytes [13]. The observed higher titres of malaria-specific antibodies in the Fulani compared to the Dogon for IgG1-IgG3 but not for IgG4 are in concordance with previous studies [14-16]. These differences were still significant when correcting for age (not listed). Regarding IgG3 levels, one interesting observation was made when dividing the children into infected and uninfected individuals. The infected Fulani had lower titres of IgG3 antibodies than uninfected children. This is surprising since IgG3 is an isotype that has previously been suggested to play an important role in the protection against malaria [13]. One reason for the lower levels seen in Fulani could be that these antibodies have formed immune-complexes and thus are no longer detectable in the circulation. However, after correcting for age this difference was not statistically significant between the two groups anymore (not listed). The reason for this is not know but might be due to the different age distributions in the two groups (the infected Fulani children included the youngest children in the study) as well as the small sample size. Further studies are needed to clarify this.

The result showed that the titres of malaria-specific IgG2 and IgG4 antibodies were relatively low in both Fulani and Dogon. These antibodies are thought to inhibit the protective effects mediated by the cytophilic antibodies (IgG1 and IgG3) and could thereby potentially inhibit their neutralizing effect on the parasite. However, the role of IgG2 antibody has shown somewhat conflicting results and recent studies have indicated that this antibody may have a protective role against *P. falciparum *malaria in individuals carrying a certain Fcγ receptor subtype [15]. Furthermore, it has also been shown that high levels of IgG2 in combination with low levels of IgG4 antibodies are associated with protection against malaria [41]. There are also studies indicating that it is not the relative amount of these malaria-specific antibodies that is important, but rather the balance between the cytophilic and the non-cytophilic antibodies that determines the outcome during an infection [42].

## Conclusion

Taken together, the results from this study demonstrate that there are marked differences between Fulani and Dogon immune responses to *P. falciparum *infection and that these differences are evident at an early age. The Fulani were shown to have higher levels of all tested inflammatory cytokines compared to the Dogon children, especially levels of IFN-γ, a cytokine that is crucial in determining the outcome of an infection. When children were further subdivided into infected and uninfected individuals, infected Dogon children had significantly lower levels of RANTES compared to uninfected Dogon, while significantly higher levels of MIG and IP-10 and also higher levels of MCP-1. Such patterns of differences in chemokine levels between infected and uninfected individuals were not evident in Fulani children whose levels remained unchanged upon an infection with *P. falciparum*. In addition, the results showed that the Fulani children had higher titres of malaria-specific antibodies compared to the Dogon children. This was true for levels of IgG1 and IgG3 antibodies, which have been shown previously to be involved in protection against malaria. The findings presented here suggests that there are marked differences in the cytokine and chemokine levels as well as in malaria-specific antibody titres between Fulani and Dogon that can be found at childhood age in these ethnic groups. Such difference might be important for the relative protection against malaria seen in the Fulani as compared to the Dogon. However, due to the small sample size in this study a prospective evaluation of a larger cohort of patients is required to confirm the results presented here. Further investigations are needed to elucidate the underlying mechanisms responsible for Fulani's relative resistance against malaria and hopefully, such insights may help in control strategies in malaria-endemic areas.

## Abbreviations

APC: antigen presenting cell; CBA: cytometric-bead array; DC: dendritic cell; FACS: fluorescent activated cell sorter; IFN: interferon; Ig: immunolglobulin; IL: interleukin; IP: IFN-gamma-inducible protein; MCP: monocyte chemotactic protein; MIG: monokine-induced by IFN-γ; PE: phycoeryhtrin; P. falciparum: *Plasmodium falciparum*; RANTES: regulated on activation normal T cell expressed and secreted; TLR: toll like receptor; TNF: tumor necrosis factor.

## Competing interests

The authors declare that they have no competing interests.

## Authors' contributions

PG, CA, VD, BT, AD, OD and MTB conceived, designed and coordinated the study. PG and CA participated in the sample collection and processing. SB and MTB designed and supervised the immunoassays. SB carried out the immunoassays. SB and JP performed statistical analysis. SB drafted the first version of the manuscript. All authors read and approved the final manuscript.
